# Lateral Tension Upper Body Lift: “Zip-Shark” Technique as Novel Procedure

**DOI:** 10.1007/s00266-025-05233-9

**Published:** 2025-09-18

**Authors:** Evgeni Vanyov Sharkov, Irina Georgieva Sharkova, Dimitar Plamenov Simeonov

**Affiliations:** Medical Center V-Derm Clinic, Sofia, Bulgaria

**Keywords:** Lateral body lift, Zip-shark, Upper body lift, Massive weight loss, Secondary procedure, Body contouring

## Abstract

**Introduction:**

The lateral tension upper body lift (Zip-Shark) is mainly used in patients after massive weight loss, predominantly as a second or third stage in a body contouring plan. This technique is a surgical procedure aimed at removing excess skin and subcutaneous fat from the chest and flanks.

**Materials and Methods:**

A retrospective analysis was conducted on 12 patients who underwent lateral body lift. Demographic and surgical data were collected and analyzed for potential factors influencing outcome.

**Results:**

In this group of 12 patients, 10 were female and two were male. In the subjective satisfaction score (1 representing the lowest satisfaction score and 5 representing the highest score) measured 12 months after surgery, 10 patients indicated the maximum value of the scale. Only two indicated the levels of satisfaction 4. The mean improvement of the pinch test was calculated as 7 cm in the 12^th^ postoperative month. Among the 12 patients included in this subgroup analysis, four (33%) experienced minor complications, all of which resolved without requiring surgical intervention. Notably, all four of these patients had a history of massive weight loss, and three of them hadn’t undergone simultaneous liposuction. Female patients ranged in age from 33 to 62 years, with an average age of 45 years and an average BMI of 25. All female patients underwent lateral body lift as an extended L-type brachioplasty. The two male patients were aged 28 and 50 and had an average BMI of 25. The most common adverse event was wound dehiscence in the armpit area, managed successfully with secondary intention healing.

**Conclusion:**

This type of procedure has been used in male and female patients. In most of the female cases, bra-line back lift would provide similar results, and scar could be hidden under the bra. Nevertheless in most of the massive weight loss cases, including females, skin excess along the horizontal access could be targeted more efficiently by using the lateral chest excision. Lateral body lift (Zip-Shark) has been described as another additional procedure of choice to achieve simultaneous lifting of the soft tissues in the back, abdomen, pectoral area, and upper arms.

**Level of Evidence V:**

This journal requires that authors assign a level of evidence to each article. For a full description of these Evidence-Based Medicine ratings, please refer to the Table of Contents or the online Instructions to Authors www.springer.com/00266.

## Introduction

Lateral tension upper body lift (“Zip-Shark”) is a surgical procedure aimed at removing excess soft tissue from the area of the chest, flanks, and upper arms in the cases of simultaneous brachioplasty technique [1]. The rationale for the lateral body lift procedure is to achieve simultaneous lifting in the upper body, including upper arms in some cases, and to improve the contouring in the abdominal area and the area of the flanks, when this is needed. In comparison with all other upper body lift techniques “Zip-Shark” lateral body lift focuses on skin excess along the horizontal axis. Existing upper body lift procedures focus mainly on the vertical soft tissue excess, rather than the horizontal one. This type of procedure has been executed as secondary technique in massive weight loss patients. It has been executed as a primary technique mainly in cases with an L-type brachioplasty.

**Fig. 1:**
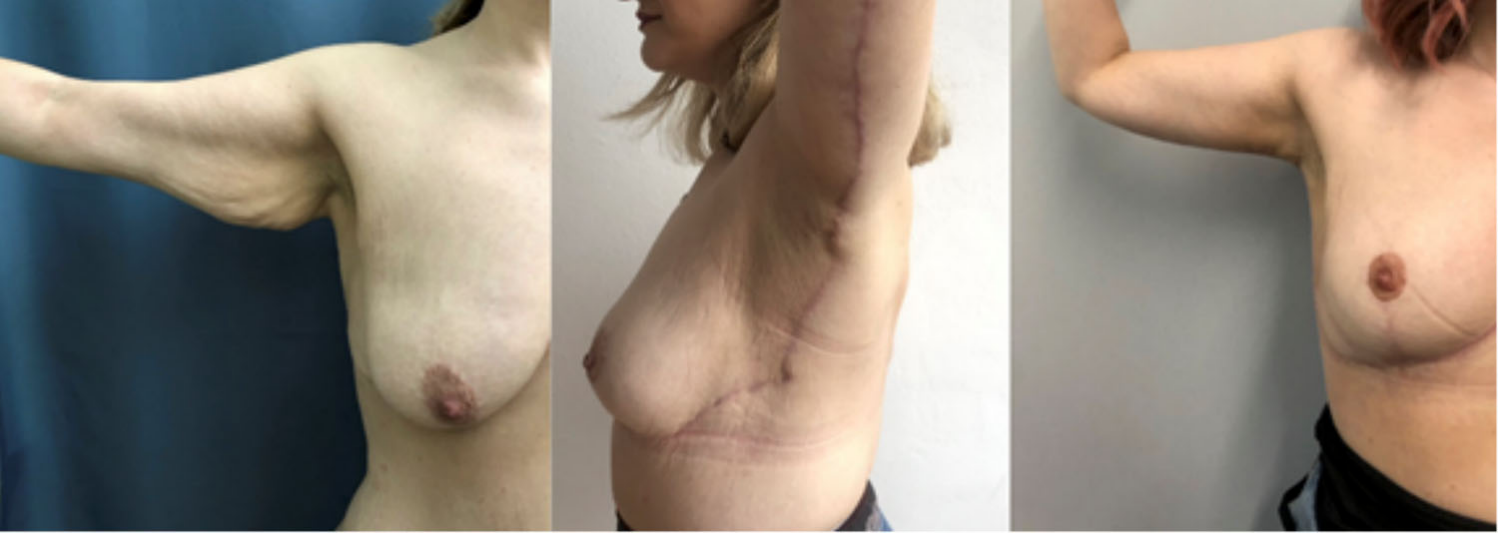
Single-stage L-type brachioplasty, with a scar positioned on the posterior surface of the upper arm, combined with an inverted-T mastopexy with mobilization of the subcutaneous soft tissues from the lateral surface of the chest, before and 1 year after the procedure

**Fig. 2:**
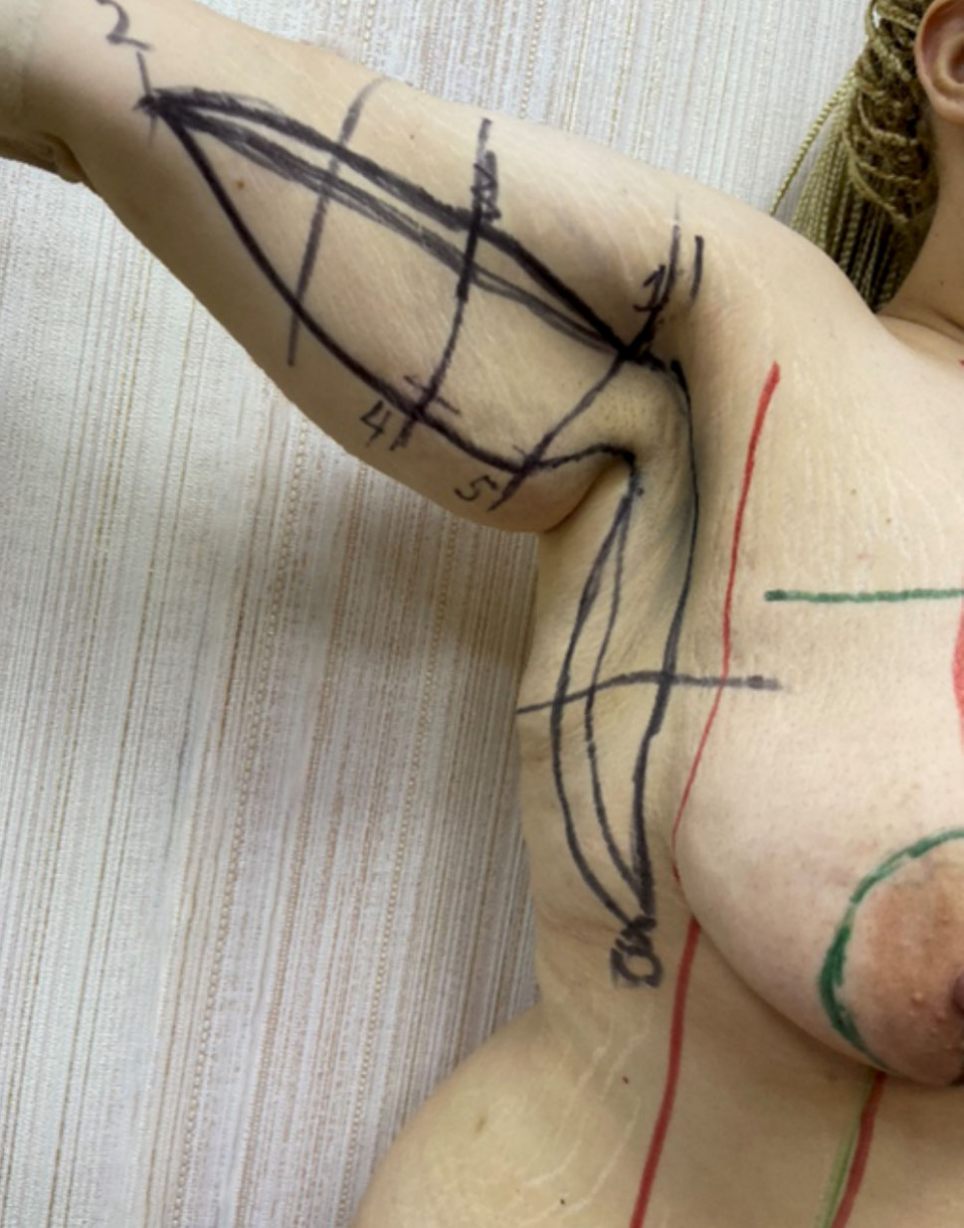
Preoperative markings with positioning of the scar in the bicipital groove. In the specified case, an L-type brachioplasty was performed, as the “arm” on the lateral surface was marked in such a way that the anterior incision is 1.5 cm posterior to the lateral edge of pectoralis major sulcus, which makes this part of scar also “invisible” when viewed in neutral arm position

**Fig. 3:**
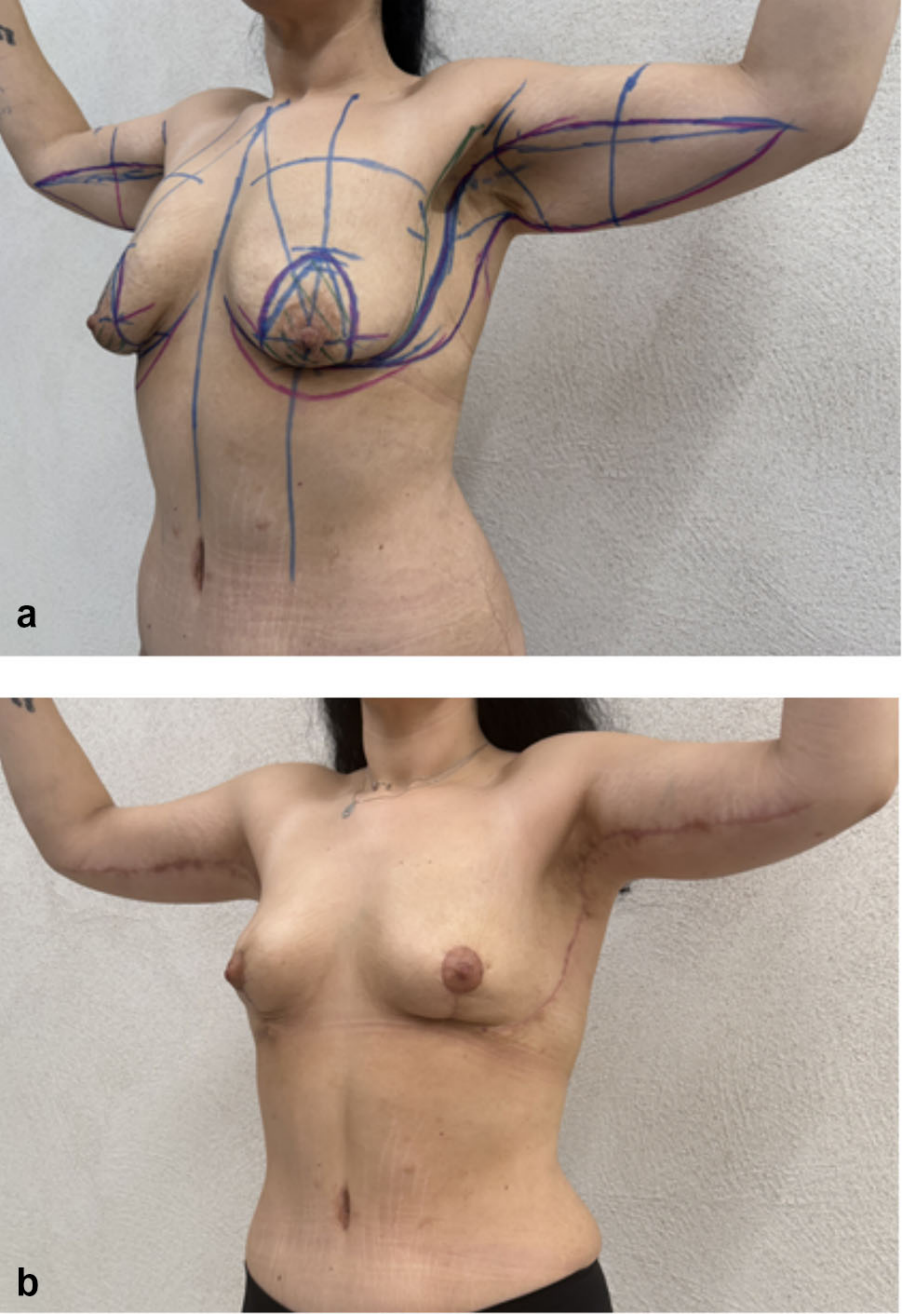
**a**, **b** Before and 3 months after J-torsoplasty with mastopexy in a single-stage massive weight loss female patient

**Fig. 4:**
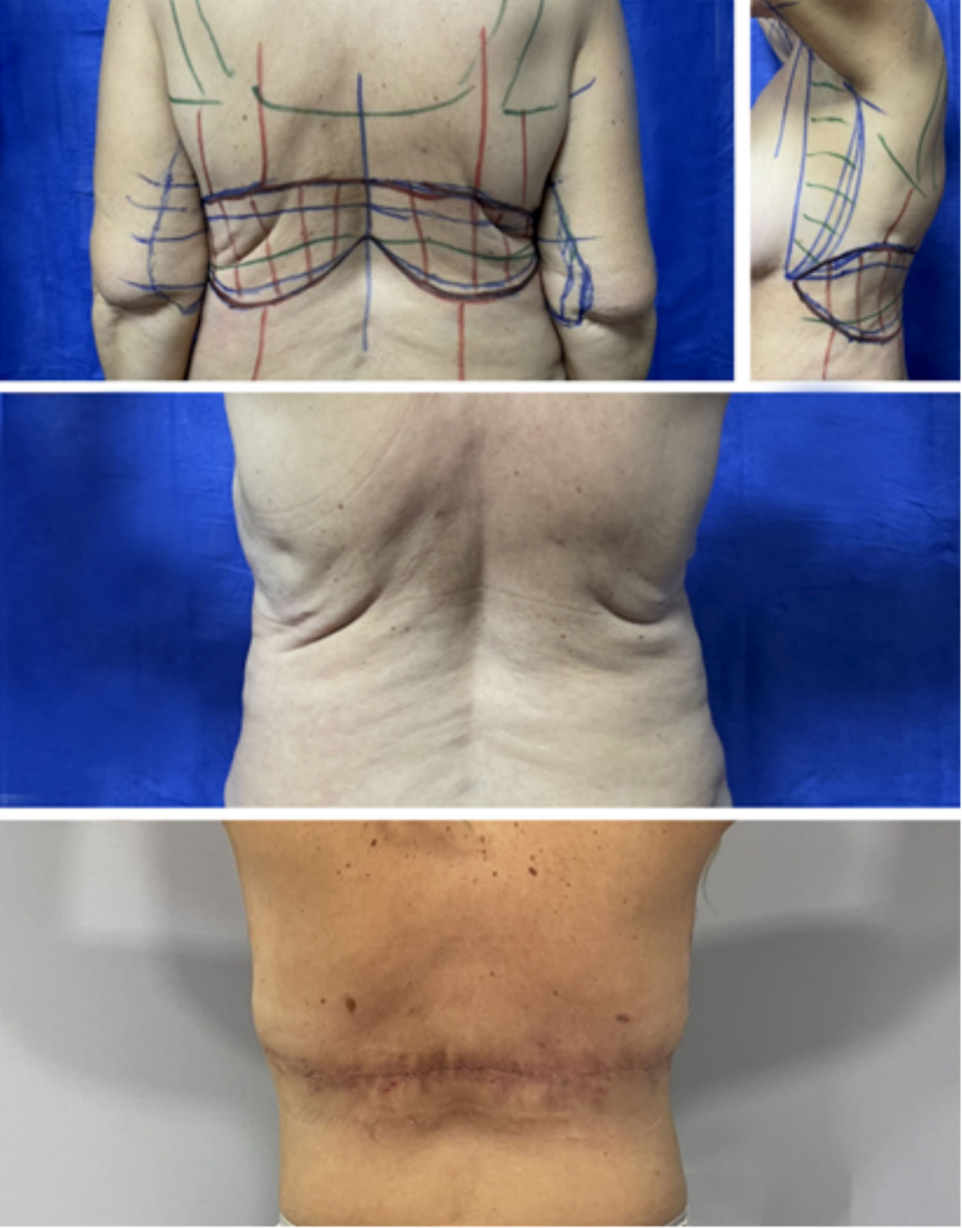
Bra-line back lift in combination with L-type brachioplasty in a 54-year-old female patient with previous liposuction in the area of the back performed in another clinic more than 5 years ago. Demonstration of the preoperative markings and early postoperative results with the corresponding position of the postoperative scar—30th postoperative day

Similar techniques have been described previously. L-type brachioplasty with posterior positioning of the scar in the upper arm area has been an excellent solution in massive weight loss patients. In female patients, this could be combined in a single-stage procedure with lateral chest soft tissue repositioning when performed together with a breast lift surgery [2] (Fig. 1). The authors’ preferred technique in L-type brachioplasties has been to position the scar in the bicipital groove and proceed in the armpit area with an advancement flap rather than a Z-plasty technique [3] (Fig. 2). J-torsoplasty has been also described as a possible technique in this area [4] (Fig. 3 a, b). Transverse upper body lift [5] and upper body lift with lateral excision [6, 7] are procedures of choice, when contouring the upper and lower back area. In these cases, the postoperative scar has been positioned the same way as in the traditional bra-line back lift procedure (Fig. 4).

## Methods

**Fig. 5:**
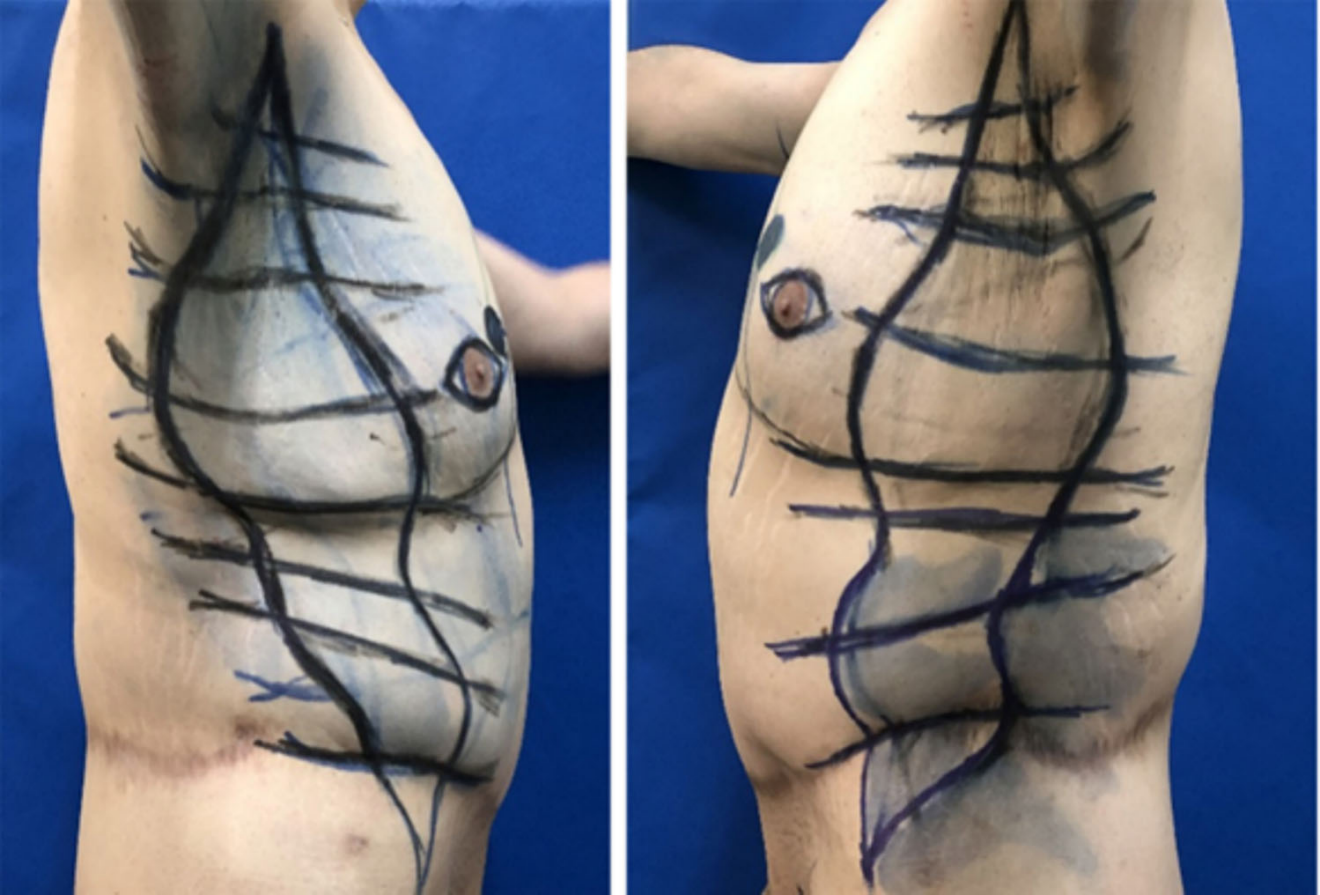
Patient after massive weight loss and previous belt lipectomy. Preoperative markings in a wavy ellipse shape; markings of the future position of the nipple–areola complex (NAC)

**Fig. 6:**
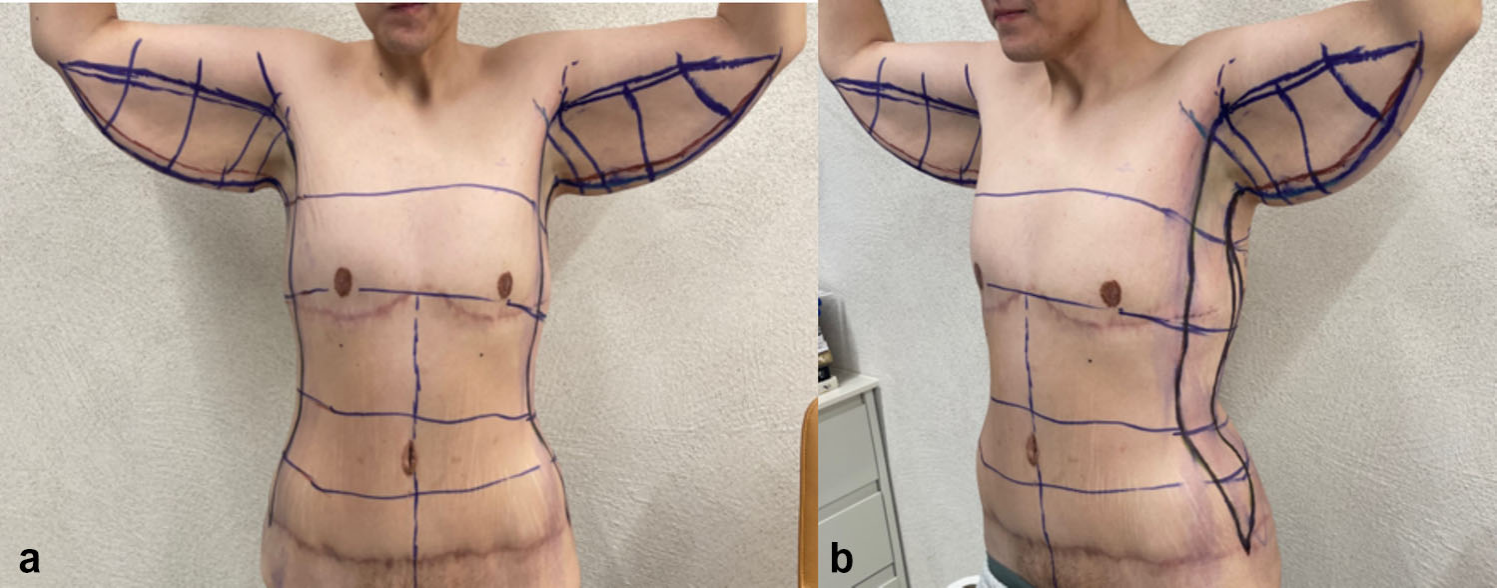
**a**, **b** Six months after upper body lift with extended abdominoplasty in a massive weight loss male patient. Preoperative markings of lateral body lift as the extension of L-type brachioplasty. Markings are the key factor to prevent lateral displacement of the NAC, when possible

**Fig. 7:**
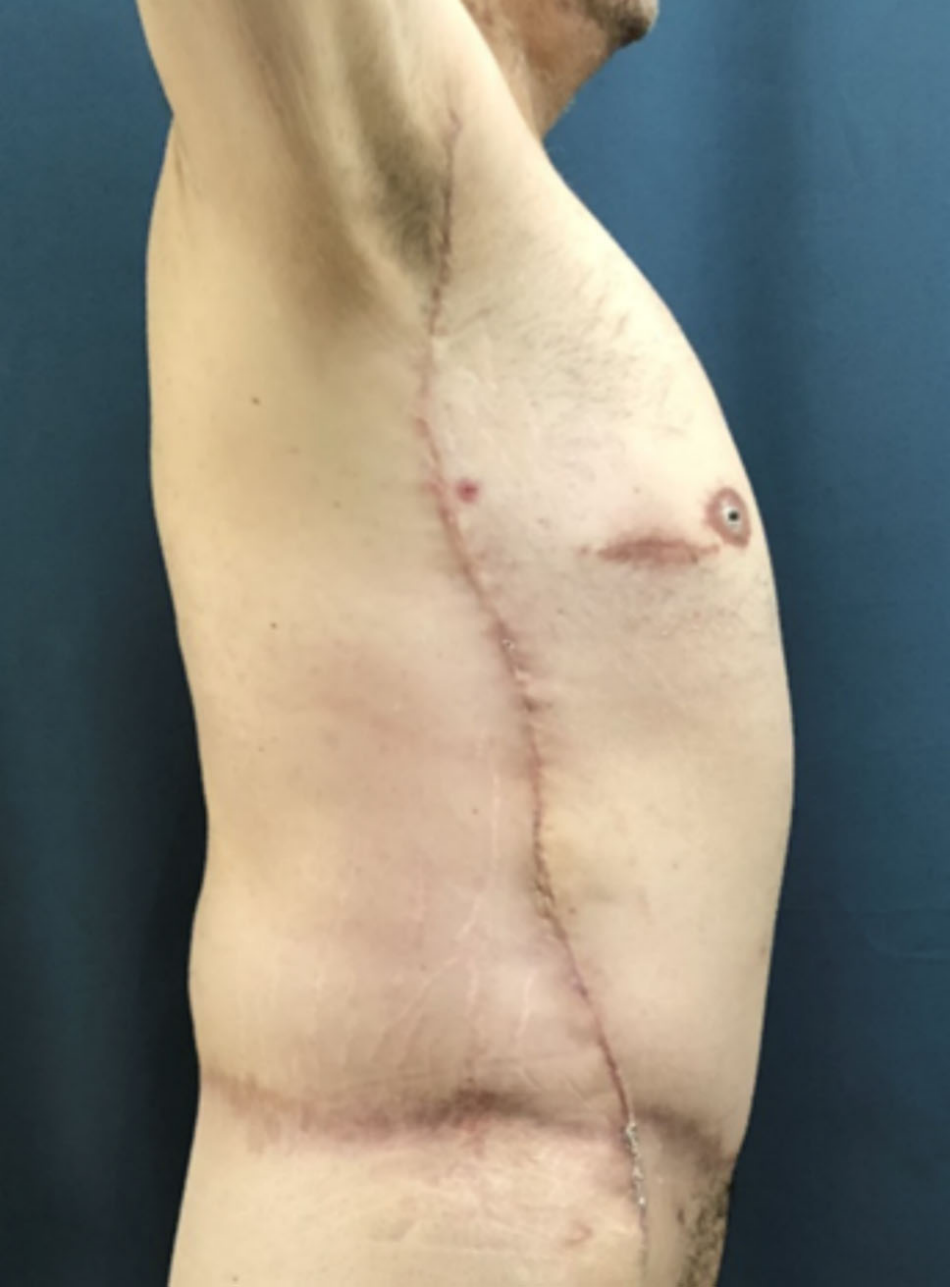
Third postoperative month in a post-bariatric patient with the previous belt lipectomy procedure

**Fig. 8:**
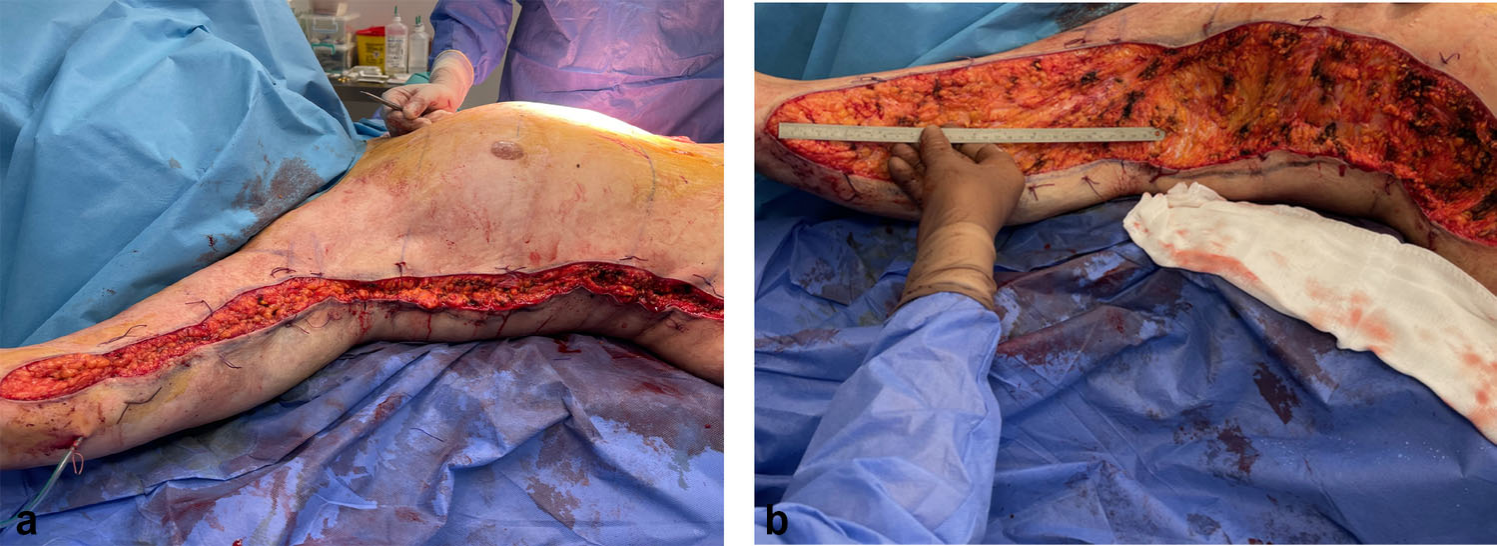
**a**, **b** Intra-operative view of lateral body lift (Zip-Shark technique) in combination with L-type brachioplasty

**Fig. 9:**
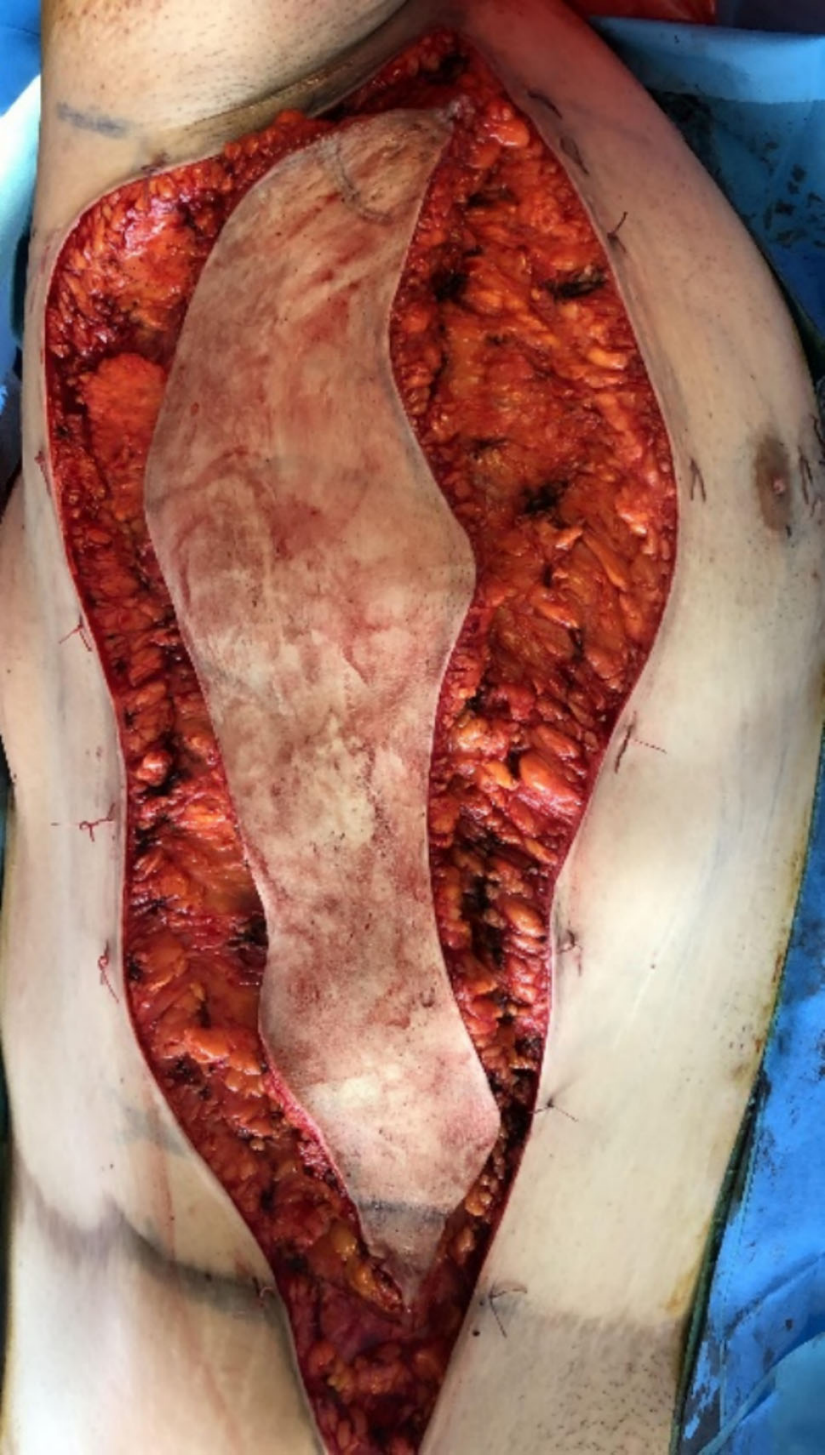
View of the removed cutaneous–subcutaneous excess during a lateral tension upper body lift procedure. The resulting defect is 20 cm wide and 60 cm long

**Fig. 10:**
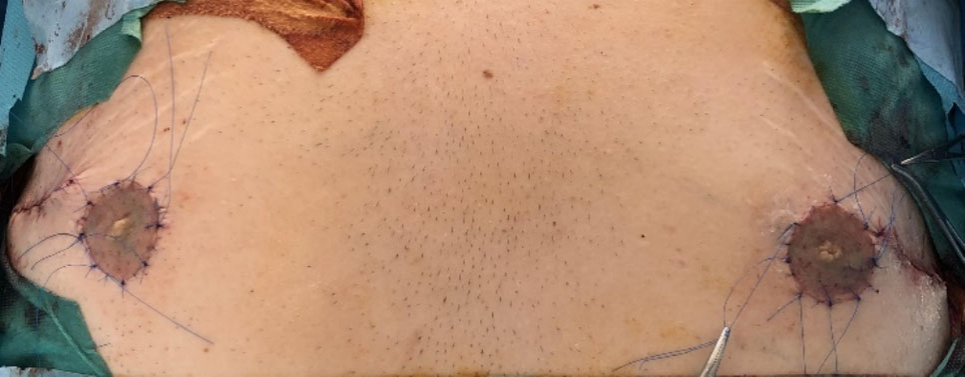
Repositioned NAC in the form of ACL as the final stage of the intervention (when this is needed)

**Fig. 11:**
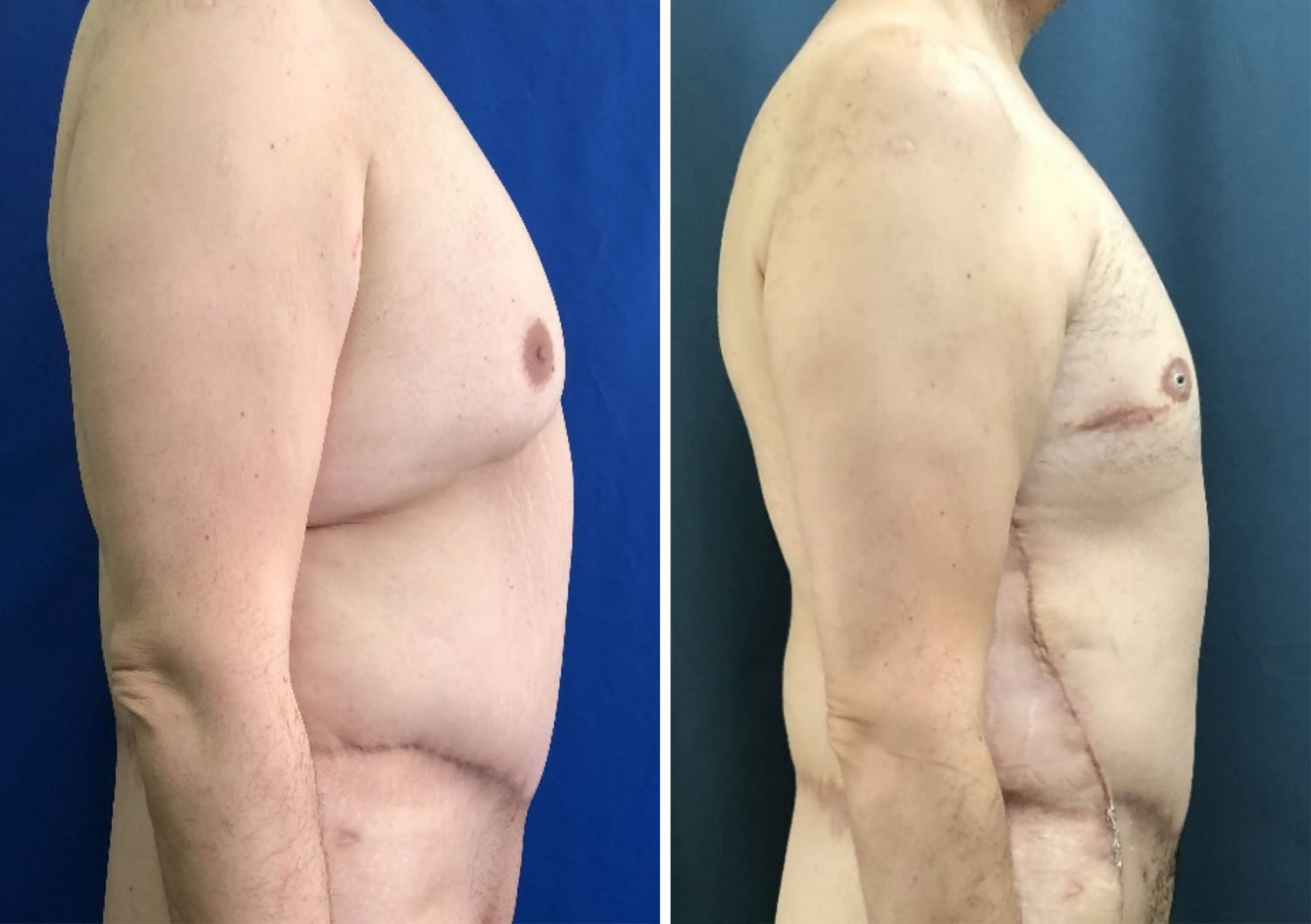
Patient with massive weight loss after bariatric surgery. Photograph on the left indicates 1 year after belt lipectomy; right photograph shows the early result in the third postoperative month after lateral tension upper body lift

**Fig. 12:**
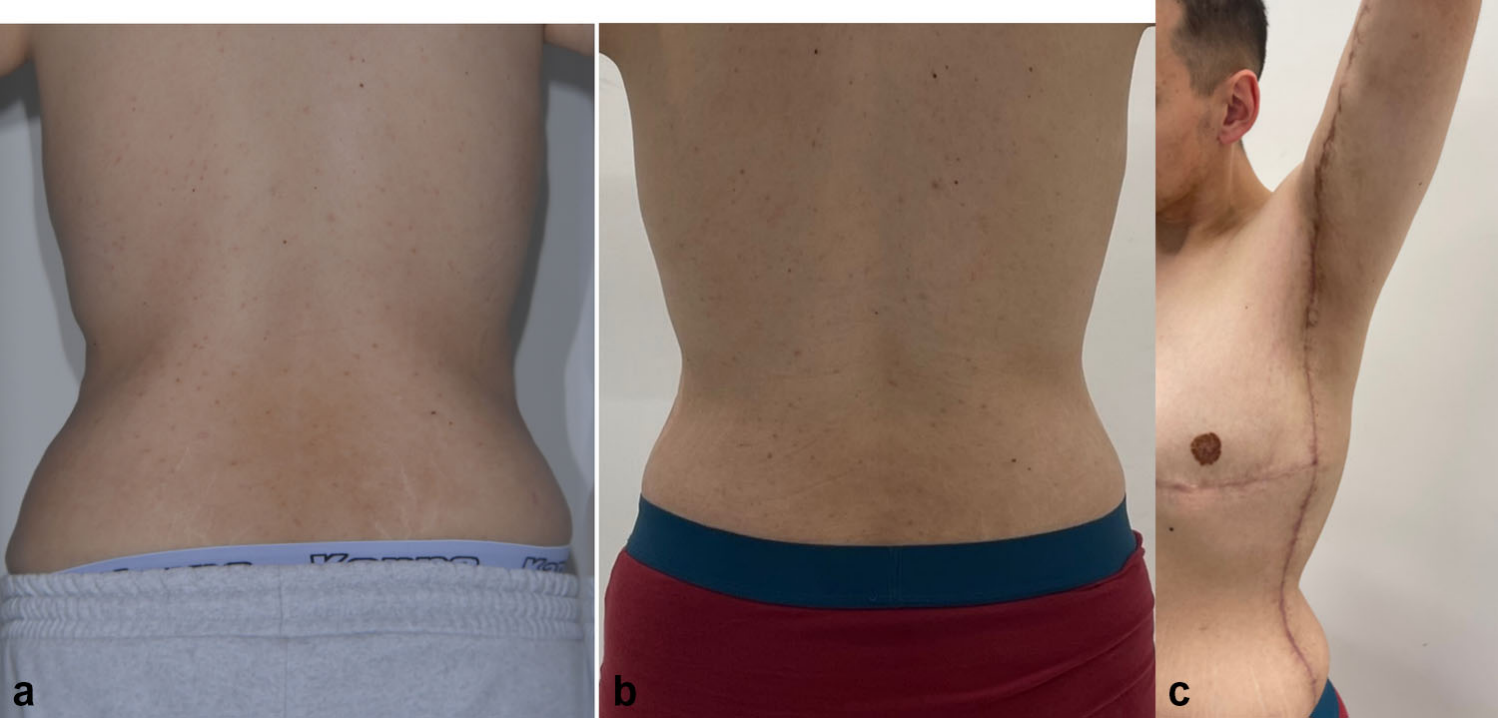
**a**, **b**, **c** Six months after lateral body lift. Procedure was done 6 months after extended abdominoplasty with upper body lift (with NAC transferred as a free skin graft). “Zip-shark” has been performed as elongation of the L-type brachioplasty. Postoperative scar is well positioned on the lateral body area, under the arms. Lifting of the skin on the back area has been achieved, and NAC has maintained its position, without lateral displacement

Lateral body lift has been conducted in 12 patients. The excision volume is determined by means of a “pinch test,” marking a wavy ellipse on the lateral surface of the chest extending from the fossa axillaris to below the level of the iliac crest. The wavy shape aims to prevent the possible hypertrophic nature of the postoperative scar. A key factor is the determination of the future position of the nipple–areola complex (NAC), which may be displaced laterally in the lateral pull in the breast area during the closure of the surgical defect (Figs. 5, 6, 7). It is essential to discuss with the patient both the position and the length and possible hypertrophic evolution of the postoperative scar. This type of procedure could be also combined with contouring in the area of the arms. In these cases, the ellipse on the lateral chest wall could be presented as further elongation caudally of an L-type of brachioplasty (Fig. 6 a, b). Under general anesthesia, after positioning the patient in a side position on the surgical table, a cutaneous–subcutaneous excision of the segmental-closure resection type is performed—starting with the arms when the procedure has been performed as extension of L-type brachioplasty (Fig. 8 a, b). The excision is made above the thoracic fascia in the lateral chest area and, accordingly, above the fascia in the flanks. After a thorough hemostasis, undermining both posteriorly and anteriorly has been done—a key factor is the predominant undermining toward the back (up to 4–5 cm) and less undermining of the anterior flap (around 2 cm), and thus, lateral displacement of the NAC would be less possible. Deep fixation fascial sutures with 0/0 PDS are placed, after which the surgical defect is closed in layers, using 2/0 and 3/0 PDS and 4/0 Monocryl suture (Fig. 9). The author uses Redon drainage, which is brought out at a declivous location. Then, the patient is repositioned, and a similar approach is applied to the contralateral site. In position of lying on one’s back, a repositioning of NAC is performed in the form of ACL, when needed (Fig. 10). In cases where simultaneous liposuction has been performed, this has been executed before the excision. The author uses the standard Klein solution. Liposuction (PAL—power-assisted liposuction) has been conducted with N4 Mercedes-type cannula in deep plane around the excision site and superficially in the area to be excised, thus alleviating the following excision and undermining (Fig. 11, Fig. 12).

All 12 patients underwent the same postoperative protocol including drains, compression garments, and scar therapy.

## Results

A retrospective analysis was conducted on 12 patients who underwent a lateral body lift procedure to remove excess skin and subcutaneous fat from the area of the chest, flanks, and upper arms. The average age was 45 years, and the average body mass index (BMI) was 26. In this group of 12 patients, 10 were female and two were male. Female patients ranged in age from 33 to 62 years, with an average age of 45 years and an average BMI of 25. Among them, two patients (20%) experienced complications. Complications occurred in patients aged 46 and 47, with BMIs of 24 and 27, respectively. Both patients had a history of massive weight loss. All female patients underwent lateral body lift as an extended L-type brachioplasty. The two male patients were aged 28 and 50 and had an average BMI of 25. One of them experienced lateral displacement of the nipple–areola complex, and the other had undertreatment of the upper arm area. Overall, among the 12 patients included in this subgroup analysis, four (33%) experienced minor complications without requiring second surgical intervention. Notably, all four of these patients had a history of massive weight loss, and three of them had not undergone simultaneous liposuction. The most common adverse event was wound dehiscence in the armpit area, managed successfully with secondary intention healing (Table 1).

**Table 1:** Complication overview

Patient	Characteristics	Massive WL	Liposuction	Complication Type	Outcome
1	F, 47	Yes	No	Wound dehiscence in the armpit area - secondary healing	No revisional surgery needed
2	F, 46	No	No	Wound dehiscence in the armpit area - secondady healing	No revisional surgery needed
3	M, 50	No	No	Latral displacement of the nipple areola complex	Free nipple-areola complex transfer was performed during the surgery
4	M, 28	Yes	Yes	Insufficient contouring (upper arm)	Secondary surgery would be recommended in the area of the arm as an isolated brachioplasty

Excised soft tissue ranged from 45 to 55 cm in length and 15 to 20 cm in width, with an average of 47.5 cm in length and 17.5 cm in width. Regarding the weight of the excised tissue, it was calculated that the average amount was approximately 400g per side.

Despite the relatively small number of individuals analyzed in this article, a clear conclusion can be drawn about the improvement in skin elasticity when measured before and 12 months after the procedure (Table 2).

**Table 2:** Pinch test before and 12 months after the procedure

Pinch test		
	Baseline measurement *	After 12 months*
N	12	12
Mean	10.4710	3.8975
Minimum	7	3
Maximum	15	5

*pinch test in centimeters

In the subjective satisfaction score (1 representing the lowest satisfaction score and 5 representing the highest score) measured 12 months after surgery, almost all indicated the maximum value of the scale (Table 3). Only two indicated the level of satisfaction 4—one patient with undertreatment in the area of the upper arm and one patient with secondary healing in the armpit. These data are an indicator of a very high level of patient satisfaction.

**Table 3:** Patient satisfaction level after 12 months

Patient subjective experiences after 12 months	
	Patient satisfaction
N	12
Mean	4.833
Median	5
Std. error of mean	0.05
Minimum	4
Maximum	5

*1 represents the lowest satisfaction score and 5 represents the highest score

Possible side effects related to prolonged anesthesia time due to side positioning of the patients have not been reported. The supine position could reduce furthermore the disadvantages of side positioning, and the authors believe that this could lead to insufficient contouring due to limitations of needed undermining of the soft tissues.

In one case, a secondary lateral body lift procedure done in massive weight loss male patient, free NAC transfer has been performed during the surgery due to lateral displacement of the same. A thorough preoperative marking should be taken into consideration to reduce possible complication of this type.

**Fig. 13:**
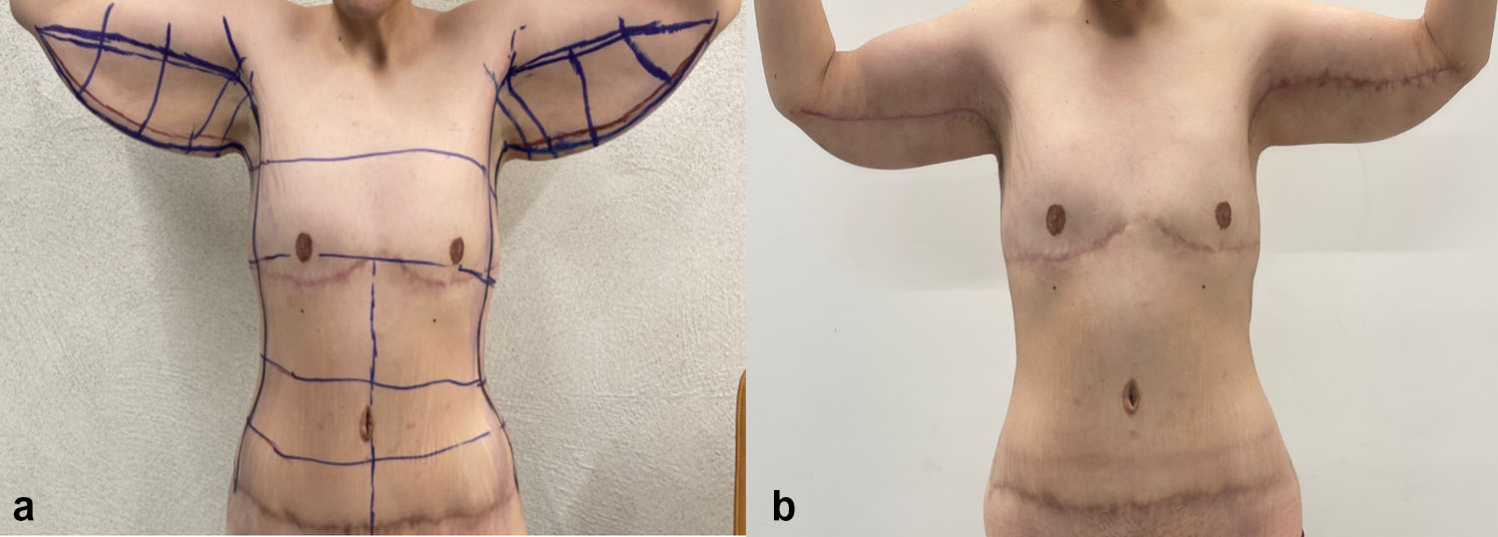
**a**, **b** Twelve months after lateral body lift. Procedure was done 6 months after extended abdominoplasty with upper body lift (with NAC transferred as free skin graft). “Zip-shark” has been performed as elongation of the L-type brachioplasty. NAC has maintained its position, without lateral displacement

In one case, a secondary lateral body lift procedure done as an elongation of L-type brachioplasty, insufficient contouring in the upper arm has been reported as undesirable outcome (Fig. 13a, b). Secondary surgery would be recommended in the arm as isolated brachioplasty.

Additional complications such as hematomas, infection, necrosis, and hypertrophic scarring have not been reported.

## Discussion

Lateral body lift differs from all other upper body lift techniques by focusing on the skin excess along the horizontal axis. Previously described upper body lift procedures focus mainly on the vertical soft tissue excess, rather than the horizontal one, which is the main target in the lateral body lift.

This type of procedure has been executed as a secondary technique in massive weight loss patients. It has been executed as a primary technique mainly in cases with L-type brachioplasty.

Determination of the future position of the nipple–areola complex (NAC) is one of the key factors to success in this type of surgical technique. It may be displaced laterally in the lateral pull of the chest/breast area during the closure of the surgical defect. This type of complication should never happen in female patients and could be resolved with free NAC skin graft in male patients. The author has reported one male case and has not reported any cases of lateral dislocation in female patients.

It is important to discuss with the patient about both the position and the length and the possible hypertrophic evolution of the postoperative scar.

This procedure could be easily combined with deep liposuction in neighboring areas and superficial liposuction in the area to be excised. In five patients who underwent the procedure, liposuction of the neighboring area (arms and/or flanks and/or chest area) was done in the same procedure prior to the excision. In all those five cases, the main goal of this combination has been to alleviate the following undermining of the tissues and to reduce the tension during the time of the wound closure. Only one case with combination of excision and liposuction has been reported with complication—“insufficient contouring of the area of the upper arm.” This complication cannot be related to the combination of liposuction and excision. The undertreatment of the upper arm has been reported by the author as insufficient excision and could be treated in the next stage of body contouring plan in a massive weight loss patient. Liposuction should be done in a conservative way, with small cannulas, thus preventing wound healing issues and necrosis of the flaps.

The advantages of the procedure are as follows: possible simultaneous lifting in the upper back, arms, pectoral area, and upper abdomen; targeting the skin excess along the horizontal access in a sufficient manner; two scars instead of a single visible one of the “Fleur-de-lis” technique and/or the bra-line back lift procedure—in neutral position of the body, those scars would be less visible under the arms.

Possible disadvantages are as follows: two scars—two wound healing areas; side positioning on the operating table; possible need to transfer the NAC as a free graft due to lateral dislocation; and flattening of the abdominal area.

This study has potential limitations. The research is based on a relatively small sample size. The current lack of a control group, a short follow-up period for some patients, and the absence of objective third-party assessments would suggest additional comprehensive data to be collected.

## Conclusion

Upper body lift procedures are often a second-stage massive weight loss intervention in a body contouring surgical plan. Scars should be extremely well planned in order to be less visible. A vast number of different techniques have been described over the years. Choosing the most appropriate surgical intervention means not only the best possible outcome of the directly treated area but also an additional beneficial impact on areas treated in previous stages. Lateral body lift (Zip-Shark) has been described as another additional procedure of choice to achieve simultaneous lifting of the soft tissues in the areas of the back, abdomen, chest, and upper arms (Figs. 11, 12a, b and c, 13a, b).
